# Effects of drying and wetting cycles on the transformations of extraneous inorganic N to soil microbial residues

**DOI:** 10.1038/s41598-017-09944-1

**Published:** 2017-08-25

**Authors:** Wei Zhang, Chao Liang, Jenny Kao-Kniffin, Hongbo He, Hongtu Xie, Xudong Zhang

**Affiliations:** 1 Institute of Applied Ecology, Chinese Academy of Sciences, Shenyang, 110016 China; 2 Department of Horticulture, Cornell University, Ithaca, 14853 USA; 3 National Field Observation and Research Station of Shenyang Agroecosystems, Chinese Academy of Sciences, Shenyang, 110016 China

## Abstract

The incorporation of extraneous nitrogen (N) into amino sugars (AS) could reflect the contribution of microbial residues to soil N transformation. Investigating the impact of drying-wetting (DW) on dynamics of newly-produced AS is critical because this represents microbial-driven N retention/losses in soil. A 36-day incubation of soil samples was conducted under different drying intensities, using ^15^N-labelled-(NH_4_)_2_SO_4_ as an N source together with/without glucose addition. There were multiple DW periods and they ranged from a constant moisture content treatment, to a one day drying (low-drying-intensity, LD), a two day drying (medium-drying-intensity, MD), or a three day drying event (severe-drying-intensity, SD). The immobilization of added-N was restricted in DW when available carbon was not added, thus glucose addition increased the effect of DW on the incorporation of added-N into AS. The response of total ^15^N-AS to DW varied depending on drying intensities. The MD was beneficial to the incorporation of added-N into total ^15^N-AS, while total ^15^N-AS contents were low in SD treatment. The effect of DW on contribution of bacterial and fungal residues to N transformation was also related to drying intensities. Our study indicated that DW altered microbial transformation of added-N, and the effect was drying intensity-specific, and available carbon-dependent.

## Introduction

In cropping systems, fertilizer produced by the Haber-Bosch process adds a significant source of nitrogen (N) to soils. Microbial-mediated fertilizer-N transformation processes are highly related to soil environmental conditions (such as soil moisture and temperature), and soil nutrient conditions (such as carbon (C) and N stoichiometric relationships)^1, 2^. Drying and rewetting of surface soils is a common natural process due to fluctuating moisture conditions, and it can affect microbial C and N transformations and cycling^3–6^. Dynamic evaluation of the effects of drying and wetting cycles on microbial-driven immobilization and transformation of inorganic fertilizer N (added N) is critical because these processes can influence soil N retention and losses in agroecosystems.

Drying suppresses the activity and biomass of soil microorganisms^7^ and soil microorganisms may shift community structure towards a greater proportion of fungi, since fungi are typically less affected by drought stress than bacteria^8^. However, Denef *et al*.^9^ suggested fungi were more sensitive to drying than bacteria, as they are located on the outer surfaces of aggregates. Re-wetting increases soil microbial biomass and activity due to the increased available substrates through microbial and physical processes, including lysis of living microbial cells, release of intracellular osmoregulatory organic solutes, and exposure of previously protected organic matter by soil aggregates and colloids^10, 11^. These studies about the effects of drying and wetting cycles on soil microorganisms have mainly focused on the changes in living microbial biomass, activity and its community composition^1, 12–14^. Moreover, living microbe estimate is only a snapshot of the microbial process^1^, however, the direct microbial contribution such as the contribution of fungal and bacterial residues to N transformation process under drying and wetting cycles is largely unknown.

Soil microorganisms utilize the available N for rapid biomass build-up and turnover^22^. Microbial cell walls are formed quickly during biological metabolism and accumulate in soil as an important part of microbial residues^15, 16^. Microbial residues have relatively long residence times and may constitute an important part of soil N pool^17, 18^. The dynamics of microbial residues can be assessed by soil amino sugar analysis^19, 20^. Amino sugars in soil are mainly contained in dead microbial residues, and it can serve as a storage pool of both immobilized N and stable soil organic matter^21, 22^. Amino sugar contents have been recognized as reliable indicator for the contribution of microbial residues to N accumulation and turnover due to their slower turnover rate relative to living microbial biomass^16, 22, 23^ and the differences in microbial origin^21, 24^. Among the identified amino sugars, muramic acid (MurN) originates exclusively from peptidoglycan of bacterial cell walls^21, 24^. The chitin of fungal cell walls is the major source of glucosamine (GluN) although bacterial cell walls and the exoskeletons of soil invertebrates also contribute to this pool^21, 24^. The origin of galactosamine (GalN) is uncertain^15^. Accordingly, the mass ratios of individual amino sugars (such as GluN/MurN) have been successfully used to indicate the relative contributions of fungal and bacterial residues to N turnover^21–23^. Joergensen and Wichern^25^ stated that phospholipid fatty acid (PLFA) analysis seems to have the greatest potential for providing a quantitative insight into microbial community. However, the most consistent information on the ratio of fungal to bacterial tissue can be obtained from amino sugar data. The use of the individual amino sugar is, thus, a method that can provide information regarding the microbial gross community shifts with environmental changes^20, 21, 25, 26^, and provide information on soil microbial transformation processes of added N under drying and wetting conditions.

The availability of C sources is an important factor that could increase the utilization of added N by microorganisms^22^. Drying and wetting cycles could increase available substrates through microbial and physical processes^10, 11^, and thereby may increase microbial use efficiency of added N. In addition, many studies have emphasized the importance of drying intensity on soil microbial processes^27, 28^, showing that more intense drying after wetting may result in greater stress on the microbial community^27^. However, how drying and wetting, along with different intensity levels, affect the transformation of added N to microbial residues, together with or without available C sources, are unclear. To clarify these uncertainties, it is necessary to quantify the amounts of newly synthesized amino sugars produced by microbial utilization of added N under drying and wetting conditions. However, because large amounts of amino sugars are stabilized in soil, it is essential to distinguish the newly synthesized amino sugars from the pre-existing amino sugars pool. The differentiation of the two pools can be obtained by using an established isotope-based gas chromatography/mass spectrometry (GC/MS) technique^29^.

The objective of this study was to trace the assimilation of added ^15^N-NH_4_^+^ into amino sugars under drying and wetting conditions, together with or without the addition of glucose and different drying intensities. Our hypothesis was that drying and wetting cycles may increase the incorporation of added N into microbial residues due to the increased availability of substrates during drying and wetting cycles. Moreover, we also hypothesized that addition of available C source (glucose) may enhance the effect of drying and wetting cycles on the microbial N transformation. A better understanding of the incorporation of added N into microbial residues as affected by drying and wetting cycles has significant implications for a more complete understanding of direct contribution of microbial residues to N transformation under climate change.

## Results

### Contents of amino sugars derived from added N in the drying and wetting cycles after the addition of N source

As shown in Table 1, the three individual ^15^N-amino sugar contents were quite low and even with some treatments no amino sugars were detected in the treatments with addition of (^15^NH_4_)_2_SO_4_ only. Moreover, the standard deviations of the soil ^15^N-amino sugar contents were higher in the treatments where amino sugars were measured.

**Table 1:** ^15^N-amino sugar contents during the incubations under wetting and drying treatments amended with (^15^NH_4_)_2_SO_4_ (mean ± SD).

Treatments^a^	^15^N-amino sugar contents at different treatments (mg kg^−1^ soil)					
	Wetting and drying cycles	1st	2nd	4th	6th	9th
CW	GluN	0.39 ± 0.25	2.01 ± 0.33	2.48 ± 0.47	2.71 ± 0.33	2.31 ± 0.19
	GalN	—^b^	—	—	—	—
	MurN	0.10 ± 0.05	0.17 ± 0.04	0.25 ± 0.08	0.26 ± 0.15	0.27 ± 0.07
		1st	2nd	4th	6th	9th
LD	GluN	—	—	—	—	—
	GalN	—	—	—	—	—
	MurN	0.19 ± 0.03	0.19 ± 0.07	0.40 ± 0.11	0.15 ± 0.05	0.19 ± 0.04
		1st	2nd	4th	6th	7th
MD	GluN	1.79 ± 0.56	2.29 ± 0.81	3.17 ± 1.73	2.81 ± 1.02	2.44 ± 0.66
	GalN	0.95 ± 0.79	1.16 ± 0.63	1.87 ± 0.86	1.05 ± 0.67	1.04 ± 1.06
	MurN	0.34 ± 0.15	0.55 ± 0.46	1.29 ± 1.95	0.47 ± 0.35	0.93 ± 0.11
		1st	2nd	4th	6th	
SD	GluN	—	—	—	—	
	GalN	—	—	—	—	
	MurN	—	—	—	—	

^a^CW, continuous wetting treatment; LD, low drying intensity treatment; MD, medium drying intensity treatment; SD, severe drying intensity treatment. ^b^ “—” Represents that the ^15^N incorporation was too low to be evaluated.

### Contents of amino sugars derived from added N in the drying and wetting cycles after the addition of glucose and N

#### Soil total amino sugars

**Figure 1:**
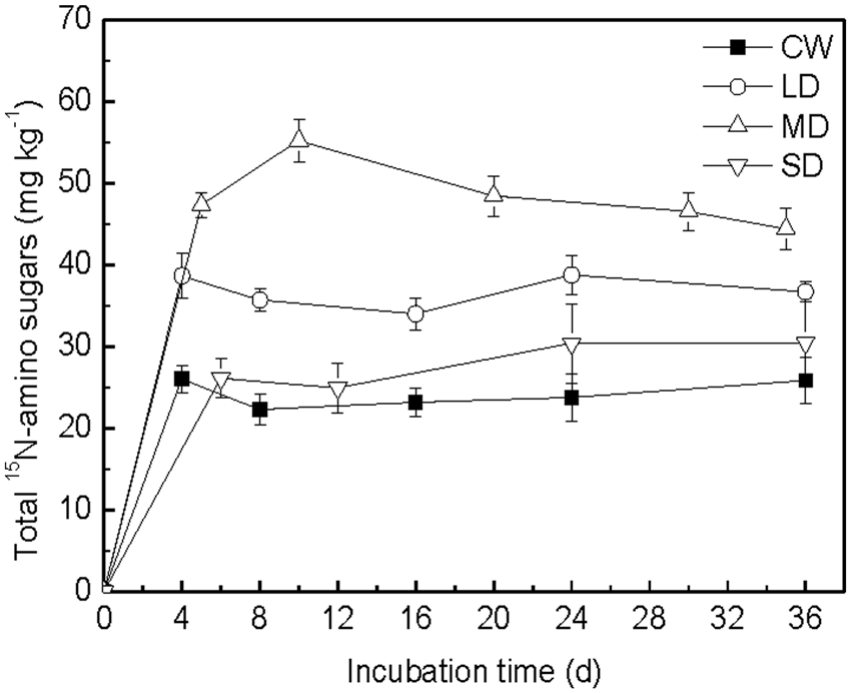
Total ^15^N-amino sugar contents during the incubations under wetting and drying treatments amended with glucose and (^15^NH_4_)_2_SO_4_. Error bars presented in the graph shows standard deviation of the means (n = 3).

The concentration of the total ^15^N-amino sugars derived from the added N increased rapidly across all treatments at the early stage of incubation, and the temporal pattern was significantly influenced by the different drying and wetting treatments (Fig. 1, *P* < 0.05). The LD and MD treatments showed higher total ^15^N-amino sugars content than the CW treatment during the whole incubation (*P* < 0.05). In the MD treatment, the total ^15^N-amino sugars content was the highest (increased by 99.6% compared to the CW treatment), but a decrease was found in the total ^15^N-amino sugars content toward the later period of incubation. No significant difference was found in the total ^15^N-amino sugars content between the CW and SD treatments (*P* > 0.05).

#### Soil individual amino sugars

**Figure 2:**
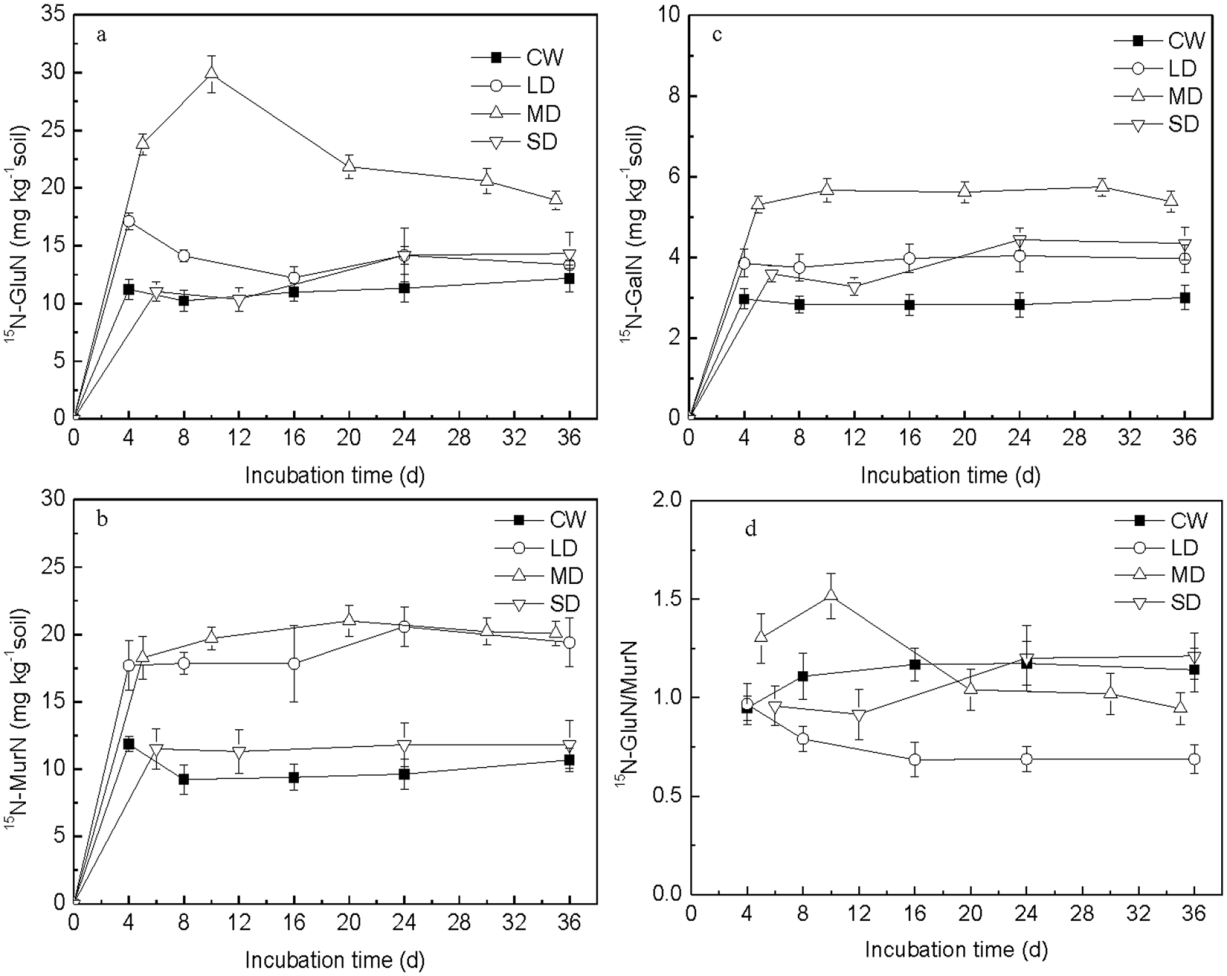
^15^N-individual amino sugar contents during the incubations under wetting and drying treatments amended with glucose and (^15^NH_4_)_2_SO_4_. (**a**) ^15^N-GluN, (**b**) ^15^N-MurN, (**c**) ^15^N-GalN and (**d**) The ratios of ^15^N-GluN/MurN. Error bars presented in the graphs show standard deviation of the means (n = 3).

The concentration of newly synthesized GluN derived from the added N (^15^N-GluN) rapidly increased with time in the CW treatment at the beginning of the incubation and reached a maximum at 4 days and then maintained this level throughout the incubation (Fig. 2a). The amounts of ^15^N-GluN were affected by the drying and wetting cycles (Fig. 2a). The content of ^15^N-GluN in the MD treatment was significantly higher than the CW, LD, and SD treatments during the entire incubation (*P* < 0.05), and it peaked (29.9 mg kg^−1^ soil) at the second cycle, followed by a declining trend toward the end of the incubation (Fig. 2a). The dynamics of ^15^N-GluN in the LD treatment exhibited a similar pattern to those of the MD treatment, and the content was significantly higher than the CW treatment at the beginning of the incubation, but no significant differences were found at the end of the incubation. The dynamics of ^15^N-GluN in the SD treatment was similar with the CW treatment with no significant difference between them (*P* > 0.05).

The concentration of newly synthesized MurN derived from the added N (^15^N-MurN), was rapidly increased in all treatments and were affected by the different drying and wetting cycles treatments (Fig. 2b). The contents of ^15^N-MurN in the LD and MD treatments were significantly higher than the contents in the CW (increased by 83.7% and 95.3%, respectively) and SD treatments during the whole incubation (*P* < 0.05). No significant difference was found in the ^15^N-MurN contents between the CW and SD treatments (*P* > 0.05).

The concentration of newly synthesized GalN derived from the added N (^15^N-GalN), increased with time over the first 4 days across all treatments (Fig. 2c). Generally, the concentration remained steady over time. The content of ^15^N-GalN in the drying and wetting treatments was significantly higher than the CW treatment (*P* < 0.05), with average increases of 35.4%, 91.5% and 35.3% in the LD, MD and SD treatments, respectively (Fig. 2c).

#### Ratios of ^15^N-GluN/MurN

In the CW treatment, the ratio of ^15^N-GluN/MurN increased at the beginning of the incubation, and then remained almost stable (Fig. 2d). In the LD treatment, the ^15^N-GluN/MurN ratio decreased gradually with time, and was lower than the ratio in the CW treatment, after the first drying and wetting cycle (decreased by 37.9%). In the MD treatment, the ratio increased at the first two drying and wetting cycles and was higher than the ratio in the CW treatment (*P* < 0.05, increased by 37.2%), then the ratio decreased with time, and became lower than the ratio in the CW treatment (decreased by 17.2%). In the SD treatment, the ratio stabilized with time at the first two cycles and was lower than the ratio in the CW treatment. After that, the ratio increased with time, and became similar to the ratio in the CW treatment over the latter part of the incubation.

### Accumulative respired CO_2_ in soil after the addition of glucose and N source

**Figure 3:**
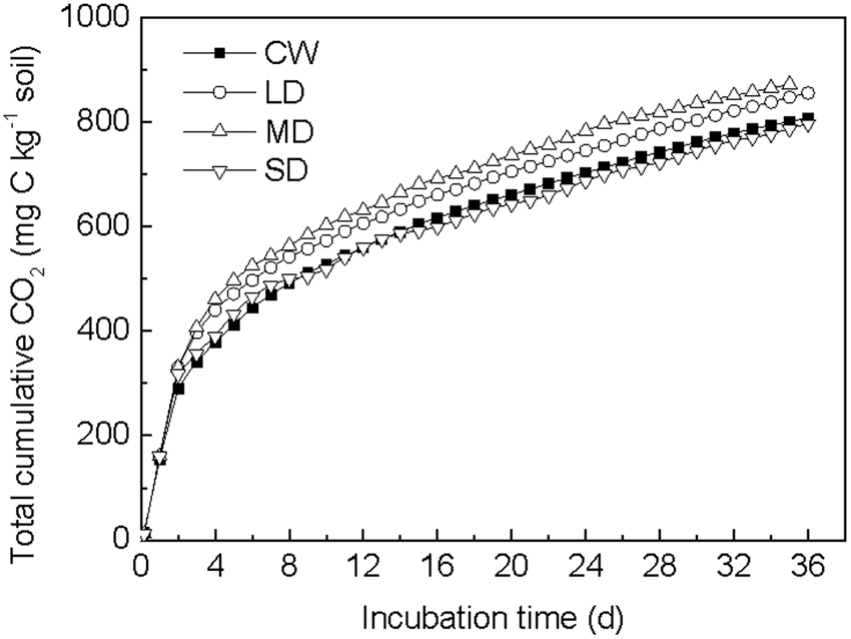
The accumulative amounts of respired CO_2_ during the incubations under wetting and drying treatments amended with glucose and (^15^NH_4_)_2_SO_4_.

The accumulative amounts of respired CO_2_ increased gradually with time in all treatments (Fig. 3). Over the entire incubation period, the total cumulative amounts of respired CO_2_ in the four treatments were as follows: MD > LD > SD ≈ CW. Compared to CW treatment, the total cumulative amounts of respired CO_2_ increased by 7.3% and 10.7% in the LD and MD treatments, respectively.

## Discussion

### Dynamics of added N incorporation into amino sugars in soils

Available substances, such as glucose, can shift soil microorganisms from dormancy to activity, increasing nutrient demand and leading to the significant utilization of added N (e.g., NH_4_^+^)^30–32^. Consequently, microbial cell walls are rapidly formed during biological metabolism and the cell wall materials accumulated in soil as an important part of microbial residues^16, 22^. This explains why the content of the ^15^N-amino sugars increased in the CW and all drying and wetting treatments when glucose and N are added simultaneously, whereas this only happened for a couple of the treatments (e.g., MD treatment) when only the N source was added. Although some studies have shown that drying and wetting cycles could increase the availability of substrates through microbial and physical processes^10, 11^, our result indicated that the immobilization of added N was severely restricted in the drying and wetting treatment when the available C source was not added to soil. Our result was in line with the findings of Formowitz *et al*.^33^, who also did not find N immobilization after soil rewetting. Our interpretation is that there could be merely rehydration of microorganisms happened after soil rewetting, rather than microbial growth, as a result no real immobilization of inorganic N by microorganisms was found^33^.

In our study, after glucose addition, the incorporation of the added N into amino sugars varied with different drying and wetting cycles, as a result of the different drying intensities. The intensity of drought periods likely plays a key role in microbial responses to the rewetting of soils, and in ecosystem C and N turnover and sequestration^2, 34^. More intense drying, as represented by the SD treatment in our study, may cause greater microbial death^28^, and lower assimilation of added N into microbial residues compared to the LD and MD treatments (Fig. 2a). The higher total ^15^N-amino sugar contents in the LD and MD treatments showed that the utilization of added N by microorganisms is enhanced under these drying and wetting treatments due to the increase of microbial activity (indicated by the increase of total cumulative amounts of respired CO_2_ in the LD and MD treatments) (Fig. 3). Previous studies also showed that soil drying can cause the death of some soil microorganisms and the survivors would reduce their physiological activity, showing a decline in adenylate energy charge^33, 35^. Consequently, after soil rewetting, the survivors could use the dead microbial cells as a source of energy and nutrients^2, 35^ and this allows for rapid regrowth of microorganisms^2^. Therefore, the increase in ^15^N-amino sugars content indicated that greater microbial contribution to the assimilation of added N in the LD and MD treatments results in a larger microbial cell residue accumulation (^15^N-amino sugars). In addition, the greatest total ^15^N-amino sugar contents in the MD treatment may be interpreted also by changes in soil pore system as affected by different drying and wetting cycles or drying intensities, which may influence the microbial processes^27^. Yao *et al*.^27^ reported that micropores volume (%) increased with decreasing drying intensities, while macropores volume (%) was greater in the more intensely dried soil. Generally, microbial activity was low in the macropores due to extreme desiccation and also was low in the water-filled micropores due to water saturation^36, 37^. Therefore, the greatest total ^15^N-amino sugar contents in the MD treatment could result from the greatest soil microbial activity due to appropriate pore volumes, which have been verified by the greatest total cumulative amounts of respired CO_2_ in the MD treatment (Fig. 3).

In addition to the response following glucose addition, the main response of ^15^N-amino sugar accumulation to wetting occurred primarily in the first drying and wetting cycles, but this response was not as evident in the subsequent ones (Fig. 1). This was likely due to the depletion of available substrates as the incubation proceeded^38^. In our study, the rate of total cumulative amounts of respired CO_2_ decreased with time, which indicated that substrate availability decreased with time. Moreover, the decrease in ^15^N-amino sugars in the MD treatments indicated a degradation dominated process. Amino sugars in soil are in a state of continuous production and degradation, defined by the relative rates of the two processes^39^. In the MD treatment, the microbial activity was the highest, thus microbes needed to degrade more substrate to maintain the higher microbial activity. As a result, the degradation rate of microbial residues was greater than the production rate. Studies have shown that soil microorganisms could decompose their own cell wall residues, in comparison to other soil organic matter fractions when available substrate is limited^15, 19^. Jawson *et al*.^40^ suggested that newly synthesized microbial cells and their metabolites could be used as available substrates to activate soil microorganisms because of the low C:N ratio, which could explain the decrease in amino sugars at the latter phase of the MD treatment. However, in general, our study showed that MD treatment was beneficial to the sequestration of added N by microorganisms. Morillas *et al*.^41^ suggested that the changes in drying and wetting cycles expected with global climate change may have a significant impact on the availability and turnover of organic and inorganic N, and that organic N forms were more buffered in the soils exposed to drying and wetting cycles. Therefore, our study may further indicate that the MD treatment is beneficial to soil N retention, because more inorganic N was transformed into organic N (such as microbial residues).

### Different responses of soil fungal and bacterial amino sugars

The ratio of fungal and bacterial amino sugars can indicate the relative contributions of fungal and bacterial residues to soil N transformation^22^. The change of ^15^N-GluN/MurN ratio in different treatments may result from differences in microbial community composition. The lower ^15^N-GluN/MurN ratio (Fig. 2d, the ratio <1.52) in the glucose and N amended treatments compared to the N amended treatments (Table 1 for MD, the ratio ranged from 2.46 to 5.98) may be due to the faster response of bacteria than fungi to the available substrate^16^. However, after glucose addition, the different responses of fungi and bacteria for extraneous N immobilization was furthermore dependent on drought intensity. In the MD treatment, the ratio of ^15^N-GluN/MurN increased in the first two drying and wetting cycles compared to the CW treatment, which showed that there was possibly a greater contribution of fungal residues to N transformation than bacterial residues. Fungi are less affected by drought stress than bacteria^8^, because fungi can remain active in soils at very low water potential^42, 43^ and they are able to extend their hyphae to reach nutrients actively down to matric potentials, at which the mobility of bacteria is considered to be negligible^44^. However, with increasing drying and wetting cycles, the ratio of ^15^N-GluN/MurN decreased and was lower than the CW treatment, which pointed toward greater contribution of bacterial residues to N transformation than fungal residues in soil under the MD treatment relative to the CW treatment in the later incubation. Zhang *et al*.^28^ showed that the fungal community appeared to be suppressed and the microbial community shifted toward bacterial dominance at the end of the drying and wetting incubation. In our study, the ^15^N-GluN content decreased later in the MD treatment (Fig. 2a), which may be due to reduced fungal growth and/or the decomposition of fungal residues. As for the lower drought stress treatment (LD), the lower ^15^N-GluN/MurN ratio in the LD treatment compared to the CW treatment, showed that there was greater contribution of bacterial residues to N transformation than fungal residues, since extraneous N was prone to accumulate in bacterial residues compared to fungal residues (Fig. 2d). Under lower drought stress, the relative growth rate of bacteria was greater than fungi, since bacteria grow fast and prefer to immobilize simple extraneous N^16^. As for the stronger drought stress treatment (SD), a greater contribution of bacterial residues to N transformation than fungal residues was found compared to the CW treatment. More drought stress as would have occurred with the SD treatment could induce more aggregate disruption, as a result, fungi were more sensitive to this drought stress than bacteria, as they are located on the outer surfaces of aggregates^9^. Therefore, the negative effect of drying on fungal biomass was greater than bacteria, which may explain the smaller contribution of fungal residues to N transformation than bacterial residues in the SD treatments in our study. However, as the drying and wetting cycles proceeded, the ^15^N-GluN/MurN ratio increased, indicated the relative contribution of fungal residues increased. Denef *et al*.^9^ found that no further aggregate breakdown was observed after further drying and wetting cycles (two drying and wetting cycles), therefore, fungi growth may be no longer affected by this drying and wetting force. To the contrary, there was a small increase of ^15^N-GluN content at the later incubation of the SD treatment (Fig. 2a), due to the greater ability of fungi than bacteria to utilize the more stable substrate^22^. Schmitt *et al*.^14^ demonstrated that changes in microbial community structure in the soil, by drying and wetting cycles, could have the potential to affect nutrient cycles, therefore, the change of relative contribution of bacterial and fungal residues to N transformation in soil due to varied drying intensities may influence soil N transformation and cycling.

## Conclusions

The results from our experiment indicated that the incorporation of extraneous N into microbial residues varied with different drying and wetting cycles. The medium drying intensity treatment enhanced the incorporation of added N into soil amino sugars, while the ^15^N-amino sugar contents were low in soil subjected to severe drying intensity. Drying and wetting cycles changed the contribution of bacterial and fungal residues to soil N transformation process, and the effect was related to the drying intensities. In addition, the immobilization of added N was severely restricted in the drying and wetting treatment when the available C source is limited or available C is not supplied to soil, thus the amendment of glucose increased the effect of drying and wetting cycles on the incorporation of added N into amino sugars. Our study also indicated that the effect of drying and wetting cycles on the microbial transformation of added N was drying intensity-specific, and available C source-dependent, which may in turn influence N retention and equilibrium processes in the soil ecosystem. Our findings are significant for the N management in crop-soil systems. Available C addition (such as crop residue return) might be the key in regulating the response of added N to soil drought under climate change. However, further research is needed to verify this mechanism under field conditions.

## Materials and Methods

### Soil sample and laboratory incubations

A bulk surface soil sample (0–20 cm), classified as an Alfisol (Typic Hapludoll)^45^, was collected from the National Field Observation and Research Station of Shenyang Agro-ecosystems, Liaoning Province, China (123°24′E, 41°31′N). The weather at the site is a temperate, humid, continental monsoon climate. The mean annual precipitation is approximately 700 mm, about 75% of which falls from May to September. The soils in the region are subjected to frequent drying and wetting events. The soil pH was 6.4 (soil:water = 1:2.5), and the SOC content is 10.2 g C kg^−1^ and the total N content is 1.06 g N kg^−1^. The soil texture is 22.6% sand, 60.7% silt, and 14.6% clay. The soil had a water holding capacity (WHC) of 0.35 kg water kg^−1^ soil. The permanent wilting point of this soil is 9% (9 g of water per 100 g of dry weight soil) (pF = 4.2, pF is the negative logarithm of soil water potential in centimeter water column height). The soil samples were air-dried and sieved (<2 mm).

Soil samples (ca. 10 g) were preincubated at 25 °C at 20% water (20 g of water per 100 g of dry weight soil, corresponding to 57% of WHC) for 1 week to stabilize microbial activity. Water content around 50% WHC is preferable for aerobic metabolism^46^. The fertilizers KH_2_PO_4_ (0.9 mg g^−1^ soil, containing 0.2 mg phosphorus (P) and 0.25 mg potassium (K) g^−1^ soil) were added at the beginning of the preincubation to ensure adequate supplies of P and K. After preincubation, 250 μl (^15^NH_4_)_2_SO_4_ (^15^N 98% atom, Cambridge Isotope Laboratories, Inc. USA) with or without glucose ((^15^NH_4_)_2_SO_4_, 18.8 mg ml^−1^; glucose, 100 mg ml^−1^) was added at the beginning of the incubation, to have 0.1 mg N and 1.0 mg C per gram soil (C:N = 10), respectively.

**Figure 4:**
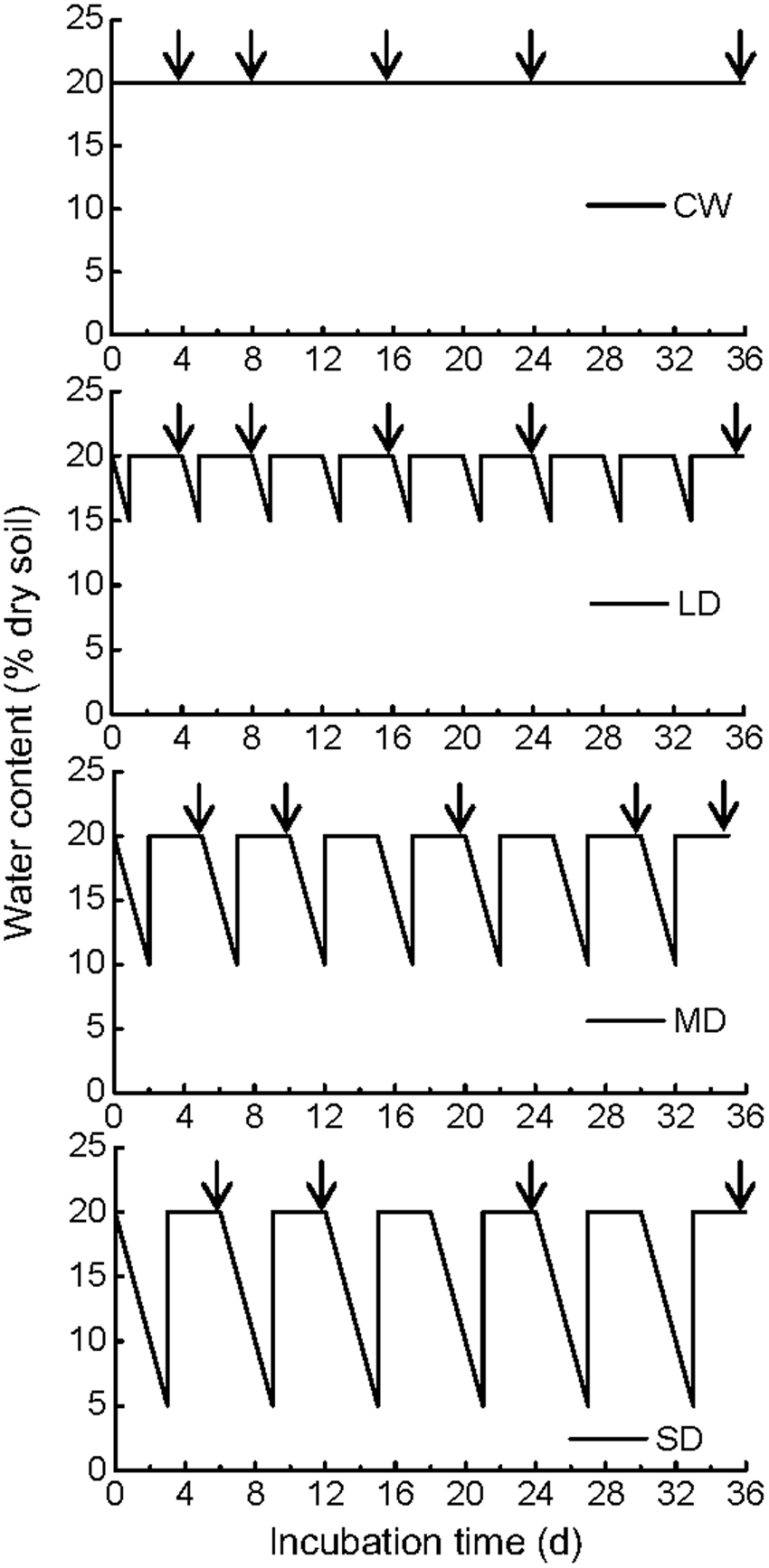
Schematic diagram of the soil moisture dynamics during the entire experiments. Arrows indicate sampling dates.

There were four treatments that were incubated for multiple drying and wetting events over a 36 day interval: (1) continuous wetting treatment (CW); (2) low drying intensity treatment (LD); (3) medium drying intensity treatment (MD); (4) severe drying intensity treatment (SD). In the CW, the soils were incubated at constant moisture content (20% water, corresponding to 57% WHC). In the drying and wetting treatments, each wetting was carried out for three days combined with different drying days, and resulting in different drying intensity. The drying time was one day in the LD, two days in the MD, and three days in the SD. By the end of the drying periods, the soils reached to 15%, 10% and 5% gravimetric water content (15 g, 10 g and 5 g of water per 100 g of dry weight soil, corresponding to 42, 28 and 14% WHC, respectively) in the LD, MD and SD treatments, respectively. The drying rate in our study can be compared to the rates of drying in other published studies^47, 48^. The number of drying and wetting cycles was different across the treatments of drying intensity during the 36-day incubation: nine cycles in LD, seven cycles in MD, and six cycles in SD. Soil samples were incubated in plastic containers (the soil bulk density is close to 1.0 g cm^−3^), which were covered with perforated plastic lids. Drying was carried out after the lid was opened for a specified period of time (1, 2, 3 d) for the particular treatment (LD, MD, SD, respectively). The soil was then rewetted to 20% water content by adding deionized water to reach a targeted weight. The soil water content was adjusted daily over the 3 days wetting periods. A schematic diagram of the soil water content during the whole incubation in each treatment was shown in Fig. 4. The experimental design is listed in Table 2. The soils were sampled at the end of each drying and wetting cycle (at the third day after rewetting in each drying and wetting cycle). Each treatment was replicated three times for each sampling time. The fresh soil samples were stored at 4 °C or air-dried samples at room temperature before analysis.

**Table 2:** The experimental treatments and sampling intervals from the measurement of soil amino sugars and respired CO_2_ during the incubation period.

Treatments^a^	Substrate	Drying length (days)	Wetting length (days)	Moisture		Frequency	Sampling intervals	
				wetting phase	drying phase			
							Soil respired CO_2_	Amino sugars^b^
CW	NH_4_^+^ or NH_4_^+^ + Glucose	0	36	20%	—	0	1 hour and 4 hour after the beginning of incubation, and then every 1 day of the whole incubation	At the same sampling day for the LD
LD	NH_4_^+^ or NH_4_^+^ + Glucose	1	3	20%	15%	9	Same as above	After 1st, 2nd, 4th, 6th and 9th wetting
MD	NH_4_^+^ or NH_4_^+^ + Glucose	2	3	20%	10%	7	Same as above	After 1st, 2nd, 4th, 6th and 7th wetting
SD	NH_4_^+^ or NH_4_^+^ + Glucose	3	3	20%	5%	6	Same as above	After 1st, 2nd, 4th and 6th wetting

^a^CW, continuous wetting treatment; LD, low drying intensity treatment; MD, medium drying intensity treatment; SD, severe drying intensity treatment. ^b^The soils were sampled at the end of each drying and wetting cycle (at the third day after rewetting in each drying and wetting cycle).

### Analysis of soil amino sugars and determination of isotope incorporation by GC/MS

Amino sugars were quantified according to Zhang and Amelung^49^. Briefly, after the soil samples were hydrolyzed with 6 M HCl for 8 hours, the solution was filtered, adjusted to pH 6.6–6.8, centrifuged (2000 × g) and freeze-dried. Then, methanol was added to remove amino sugars from the residues. Finally, the purified amino sugars were converted into aldononitrile derivatives and extracted with dichloromethane from the aqueous solution. After evaporating dichloromethane, the amino sugar derivatives were redissolved in the mixed hexane and ethyl acetate solvent (v:v = 1:1) for quantification by an Agilent 6890 GC (Agilent Tech. Co. Ltd., USA). Myo-inositol was added as an internal standard before hydrolysis and N-methylglucamine was added before derivatization as the recovery standard.

Compound-specific stable isotope analysis of individual amino sugars (GluN, GalN and MurN) was carried out with an isotope GC/MS method developed by He *et al*.^29^. The GC/MS (Finnigan Trace, Thermo Electron Co. Ltd., USA) was equipped with a quadruple MS attached chemical ionization (CI) source. The temperature and electron energy of the CI source were set at 180 °C and 70 ev, respectively. The interface temperature was 250 °C, and helium was used as a carrier gas with a flow rate set at 0.8 ml minute^−1^. The GC temperature program in CI mode was that described by He *et al*.^29^, and the split ratio was 30:1. The reaction gas was methane, and its flow was 1.5 ml minute^−1^. The ^15^N enrichment of individual amino sugars was quantified in the selected ion monitor (SIM) spectrometry mode. The mass (m/z) of the target fragments (F) as well as the corresponding F plus 1 (F + 1) mass were measured because only one N atom was observed in the amino sugar molecules. ^15^N enrichment in GluN and GalN was determined according to the intensity of m/z 206 and 207, whereas ^15^N enrichment in MurN was estimated by monitoring the intensity of m/z 264 and 265.

### Analysis of CO_2_ release

The CO_2_ release of the soil samples was determined at the 1st hour and the 4th hour at the beginning of incubation, and then every 1 day for the subsequent incubation time. At each time period, three replicates were used to measure the soil CO_2_ release. The released CO_2_ was measured by connecting a Li-COR IRGA 6262 (Li-COR Biosciences, Lincoln, NB, USA) and a mass flow meter to the outflow tube of each soil sample according to the method described by Tian *et al*.^50^, and then the flow rate and CO_2_ concentration were recorded.

### Calculation

#### CO_2_ release

The CO_2_ release rate of each soil sample was calculated from Gershenson *et al*.^51^:

1 $${{\rm{R}}}_{{\rm{r}}}=0.536\times ({{\rm{C}}}_{{\rm{c}}}\times {{\rm{R}}}_{{\rm{f}}}){/{\rm{W}}}_{{\rm{s}}}$$

where R_r_ is soil CO_2_ release rate (mg C kg^−1^ soil h^−1^), C_c_ is the recorded CO_2_ concentration in μmol CO_2_ mol^−1^, R_f_ is the recorded flow rate in mL h^−1^, and W_s_ was gram dry weight of the sample.

The total cumulative amount of respired CO_2_ was calculated by integrating the soil CO_2_ release rate with time.

#### ^15^N-labelled amino sugars

When ^15^N-labelled N was immobilized by microorganisms, the newly synthesized amino sugars were labelled and thus differentiated from the native amino sugars. Accordingly, the ^15^N enrichment of each amino sugar is expressed as atom percentage excess (APE) and calculated as follows:

2 $${\rm{APE}}=({{\rm{R}}}_{{\rm{e}}}-{{\rm{R}}}_{{\rm{c}}})/[1+({{\rm{R}}}_{{\rm{e}}}\mbox{--}{{\rm{R}}}_{{\rm{c}}})]\times 100 \% $$

where R_e_ is the isotope ratio of incubated samples and R_e_ = [A_(F+1)_/A_(F)_] (A is the integrated area of the selected ion F and F + 1). R_c_ represents the corresponding ratio obtained from original soil (before incubation) analyzed on the same GC/MS assay^29^.

Because the calculated APE represents the percentage of the isotope-containing fraction relative to the total amount of the target compound, the concentration of ^15^N-labelled amino sugar compounds can be calculated from the APE and the concentration of individual compounds, which was expressed as:

3 $${}^{15}{\rm{N}}-{\rm{AS}}={\rm{AS}}\times \mathrm{APE}/100$$

where AS is the concentration of each amino sugar determined by GC and ^15^N-AS represents the concentration of the labelled amino sugars. Total ^15^N-amino sugar concentrations (labelled amino sugars, not labelled amino sugars-N) were calculated as the sum of ^15^N-GluN, ^15^N-GalN and ^15^N-MurN (labelled GluN, GalN and MurN, not labelled GluN-N, GalN-N and MurN-N).

### Statistical analyses

A repeated measure ANOVA was performed to analyze the effects of drying intensity on soil amino sugars at different sampling time. The Tukey test was performed to assess the differences among the means of three replicates of amino sugar variables in different treatments. Significance was considered at *P* < 0.05. All statistical analyses were performed using a SPSS 13.0 software package (SPSS Inc., Chicago, USA). Figures were generated using Origin 8.0 program (Origin Lab Inc., USA).
